# [Retracted] Matrine‑induced autophagy counteracts cell apoptosis via the ERK signaling pathway in osteosarcoma cells

**DOI:** 10.3892/ol.2025.14935

**Published:** 2025-02-17

**Authors:** Kun Ma, Man-Yu Huang, Yan-Xing Guo, Guo-Qiang Hu

Oncol Lett 12: 1854–1860, 2016; DOI: 10.3892/ol.2016.4848

Following the publication of the above paper (and in light of a number of issues of concern that were raised with the Editor by independent readers, including the potentially anomalous appearance of cellular morphological data in Fig. 1D on p. 1856 and the placement of control western blots in various of the figures), the authors have requested that this paper be retracted from the Journal on account of an inability to reproduce the same experimental results.

The Editor of *Oncology Letters* has agreed with the authors’ request, and therefore this paper has been retracted from the Journal. The Editor regrets any inconvenience that has been caused to the readership on account of this retraction.

